# Extramammary Paget’s disease

**DOI:** 10.1259/bjrcr.20150261

**Published:** 2016-05-25

**Authors:** Andres Vasquez, Cristina Dominguez, Mariam Rolon

**Affiliations:** ^1^ Department of Radiology, Fundación Santa Fe de Bogotá, Bogotá, Colombia; ^2^ Department of Pathology, Fundación Santa Fe de Bogotá, Bogotá, Colombia

## Abstract

The purpose of this report is to describe an unusual case of extramammary Paget’s disease with urethral and lymph node infiltration and demonstrate the role of MRI in the pre-operative period for the assessment, management and prognosis of the disease. Although skin wrinkles on MRI may be misinterpreted based on observer's experience, it correlates well with pathology and may provide an accurate assessment before interventional therapy.

## Summary

Extramammary Paget’s disease (EMPD) is a rare form of intraepithelial skin adenocarcinoma affecting most commonly the vulva. Its incidence varies between less than 1% and 2% of vulvar malignancies,^1^ with a higher incidence in postmenopausal white females.^2^ It may also affect areas rich in apocrine glands such as the groin, thigh, buttocks, perianal region, axilla, external ear canal, eyelids, penis and scrotum.^3^ There are two types of vulvar Paget’s disease, intraepithelial adenocarcinoma arising from the vulva and perineum, and pagetoid intraepithelial spread of primary carcinoma from an adjacent area.^1^ It presents as a slowly expanding asymmetrical white and red peeling plaque on the vulva associated with pruritus (91%), pain (11%), drainage (5%) and bleeding (2%).^4^ This disease often spreads in an occult fashion with margins extending beyond the apparent lesion; therefore, the interventional treatment can be challenging,^2^ leading to positive surgical margins and frequent recurrences (30–60%).^5^ EMPD has been associated with malignancy at other sites; therefore, extensive preoperative screening has been recommended.^6^

This report describes the case of a female with non-invasive Paget’s disease of the vulva with invasive disease to urethra and lymph nodes that recurred 33 years after her initial diagnosis and management. We report the findings from an MRI, the pathological–radiological correlations and the role of MRI in the clinical management of this disease.

## Case report

This 82-year-old white female had a past medical history of a pruritic and erythematous plaque extending over her right interlabial fold. She was diagnosed with non-invasive vulvar Paget’s disease 33 years ago that was surgically treated with local excision, removing full thickness of skin involving the epidermis and dermis with a 1-cm lateral margin. Apparently, she remained asymptomatic during the next 15 years, and in 1995, a second conservative resection was preformed.

**Figure 1.**
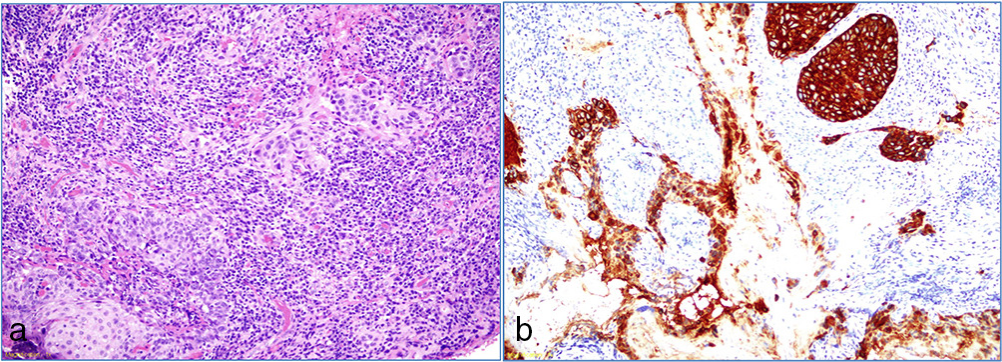
(a) Urinary meatus (haematoxylin and eosin, 20×) shows the large Paget cells with stromal tumour infiltration. (b) Intense reactivity of tumour cells for cytokeratin 7.

In 2013, she sought medical care, with a history of a 2-year vaginal discharge described as non-purulent, odourless and painless. Physical examination revealed left inguinal andenopathies of approximately 5 mm diameter, left hemivulvectomy and erythematous urethral meatus. Vulvar and urethral biopsies were positive for EMPD; immunohistochemistry was positive for cytokeratin (CK) 7, Ep-CAM/epithelial specific antigen (MOC-31) and carcinoembryonic antigen (CEA) and negative for CK20 and breast cancer antigen 2 (BRST-2). Non-invasive EMPD was found on the right labia majora, and right and left introitus. Infiltrative disease was found in the right and left lateral urinary meatus (Figure 1a), CEA+ and CK20– (Figure 1b). The vaginal wall was free of disease. Inguinal Tru-cut biopsies of adenopathies were positive for metastatic adenocarcinoma, CK7+, MOC31+, CEA+, CK20– and BRST2– (compatible with primary lesion in the vulva). Extension studies were all negative for malignancy (sigmoidoscopy, CT scan and mammography). Urethral cystoscopy showed evidence of a proliferative lesion that was not biopsied. Blood work levels were normal. Owing to the extension of the disease, the patient’s comorbidities and, most importantly, preferences, intensity-modulated radiation therapy of the pelvis was elected with a goal of completing a total of 6660 cGy.

## Imaging findings

**Figure 2.**
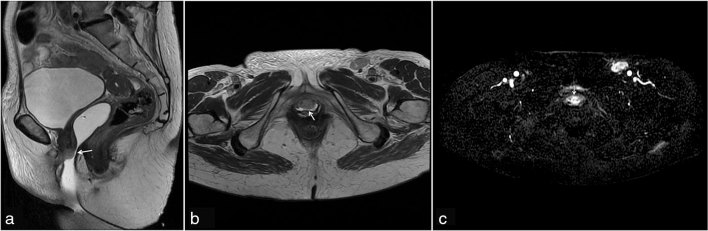
(a) Sagittal *T*_2_ weighted image with vaginal gel showing thickening of the anterior vaginal wall with a 17-mm nodular lesion (arrow) in the lower third of the vagina. (b) Axial *T*_2_ weighted image with vaginal gel showing the nodular lesion (arrow) involving the urethra. (c) The lesion showing intermediate intensity and hyperintensity on diffusion-weighted imaging.

A contrast-enhanced MRI of the pelvis was performed that revealed a 17-mm nodular lesion at the inferior third of the vagina, spreading to the urethra with irregular and asymmetrical thickening of the anterior vaginal wall. The lesion showed low-to-intermediate intensity on *T*_2_ weighted images and high intensity on diffusion-weighted imaging (Figure 2a–c). On Gd-enhanced *T*_1_ weighted images, the lesion showed homogeneous and markedly high enhancement. Multiple bilateral pelvic lymph nodes were also seen.

**Figure 3.**
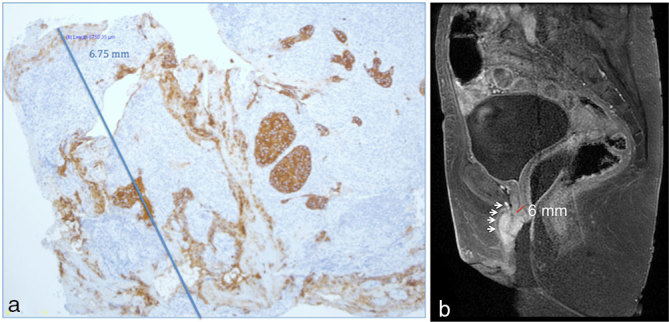
(a) Histopathological urethral depth of the Paget’s cell infiltration of 6.75mm. (b) Pelvic MRI, in-phase postcontrast sagittal image with vaginal gel reveals homogeneous and markedly high enhancement of the lesion at the anterior wall of the vagina (arrows) as well as the nodular lesion in the urethra; Gd-enhanced depth of 6.0mm.

The degree of Gd enhancement and the depth of infiltration of the urethra was evaluated. The depth of enhancement on MRI was 6.0 mm, while histopathological depth of the Paget’s cell infiltration of urethra was 6.75 mm (Figure 3). This indicates that histopathological extent of invasion correlates well with Gd-enhanced areas. Consequently, MRI is useful for pre-operative assessment because EMPD extends far beyond the clinically visible margins; it may also indicate aggressiveness of the tumour and associated malignancies.

## Discussion

EMPD is a neoplastic condition with intraepithelial infiltration of Paget’s cells. These cells extend from the epidermis to the dermis and can produce metastases. The most common site of metastases is the regional lymph node.^5^ Other sites include the bones, lungs, liver and adrenal glands. However, urethral metastases is not common. Areas of high density of apocrine glands are sites of predilection for the disease. It differs from mammary Paget’s disease, in which invasion of the dermis does not occur.^7^

Association with other malignant lesions have been found in 10–42% of cases,^5,8,9^ including colorectal, prostate, breast and cervical cancer among others. Other studies have reported 32% of the patients with invasive EMPD and 35% with *in situ* EMPD.^6^

The role of imaging is yet to be defined in patients with EMPD. MRI is useful for lesion characterization, showing the extent of disease (metastatic lymph nodes) and invasion depth, along with the detection of associated malignancies. Although there is not enough information in the literature about the imaging findings of EMPD, Akaike et al^6^ in 2013 reported three cases of EMPD that exhibited lesions with low-to-intermediate intensity on *T*_1_ and *T*_2_ weighted images, and on Gd-enhanced *T*_1_ weighted images; their markedly homogeneous enhancement findings are in accordance with our patient.

MRI may also help in the evaluation of possible associated malignancy. This requires systemic screening; however, the majority of associated malignancies tend to be located near the lesion of EMPD. Gd enhancement of the area could correlate well with the invasion depth of Paget’s cell; our case showed ureteral infiltration on Gd-enhanced *T*_1_ weighted image and was confirmed during histopathological examination of the specimen. On the other hand, EMPD may be detected on MRI performed for other malignancies.

The treatment of choice for EMPD is surgical excision with wide margins. As mentioned before, positive margins are a risk factor for recurrence, and it has also been reported that an invasion depth > 1 mm is associated with lymphatic invasion and increased tumour aggressiveness.^10^ The case reported had a depth > 5 mm in the vulvar region (high-risk patient) and pre-operative information about the extension of EMPD was highly important in order to evaluate further treatment options.

In conclusion, we described a case of EMPD and demonstrated the useful role of MRI in EMPD assessment, management and prognosis.

## Learning points

1. EMPD spreads in an occult fashion; therefore, interventional treatment can lead to positive surgical margins and high rate of recurrence.
2. EMPD has been associated with malignancy at other sites; consequently, extensive pre-operative screening is recommended.
3. MRI should be used for pre-operative assessment in patients with EMPD.
4. MRI correlates well with pathology results and may provide an accurate assessment before interventional therapy.
5. MRI is useful for lesion characterization, showing the extent of disease and invasion depth (aggressiveness), and detection of associated malignancies in patients with EMPD.

## Consent

Written informed consent was obtained from the patient’s daughter for publication of this case report, including accompanying images, since our patient passed away
